# Temperature Effect on Residual Magnetic Field of Atomic Gyroscope Magnetic Shielding System: A High-Precision Modeling Method

**DOI:** 10.3390/s26144330

**Published:** 2026-07-08

**Authors:** Yitao Chen, Junzhong Li, Shengxin Lin, Yicheng Deng, Tianshun Wang, Donghua Pan

**Affiliations:** 1 School of Electrical Engineering and Automation, Harbin Institute of Technology, Harbin 150001, China; 22b906010@stu.hit.edu.cn (Y.C.); hit_linsx@hit.edu.cn (S.L.); 2 Laboratory for Space Environment and Physical Sciences, Harbin Institute of Technology, Harbin 150001, China; junzhong.li@hit.edu.cn; 3 Quantum Engineering Research Center of CASC, Beijing 100094, China; kevin_dyc@163.com; 4 Beijing Institute of Aerospace Control Devices, Beiing 100854, China; 5 Zhengzhou Research Institute, Harbin Institute of Technology, Zhengzhou 450003, China

**Keywords:** magnetic shielding system, temperature effect, atomic gyroscope, Jiles-Atherton model, parameter identification

## Abstract

**Highlights:**

**What are the main findings?**

- An analytical model of a multi-layer cylindrical magnetic shielding system is established, with deviations from finite element method (FEM) about 5%.

**What are the implications of the main findings?**

- The proposed model provides a fast-solving theoretical alternative to computationally expensive FEM for multi-field coupling problems in atomic gyroscope magnetic shielding systems.

**Abstract:**

The residual magnetic field of the magnetic shielding system is a key factor limiting the bias stability of high-precision atomic gyroscopes. Due to the temperature dependence of hysteresis in soft magnetic materials, variations in ambient temperature can cause drift in the residual magnetic field inside the shielding cavity, thereby introducing measurement errors. Existing studies mostly rely on time-consuming finite element methods (FEM), which struggle to efficiently characterize the temperature–magnetic coupling effect. To address this issue, this paper develops a theoretical model for a fast solution. First, a static magnetic field analytical model for the multilayer cylindrical magnetic shielding system is established. Second, nonlinear magnetization theory is introduced to correct the calculation errors caused by the nonlinear variation in material permeability under weak fields. On this basis, an improved Jiles-Atherton (J-A) model incorporating a temperature correction factor is constructed to accurately characterize the magnetic field distribution inside the shielding system at different temperatures. The results demonstrate that the proposed analytical model can independently and rapidly predict the residual magnetic field distribution at different temperatures, without requiring any calibration or fitting based on FEM simulations. After accounting for hysteresis nonlinearity, the deviation of the shielding factor at the center point between the analytical model and FEM simulations is approximately 5%. The static residual magnetic field at the center point exhibits a negative correlation with temperature variation. Within the actual operating temperature range of the atomic gyroscope from −40 °C to 60 °C, the measured results agree with the model predictions regarding the temperature-dependent trend of the radial residual magnetic field. The relative deviation of the radial residual magnetic field ranges from 2.78% to 7.69%, and that of the axial residual magnetic field ranges from 7.94% to 14.47%, thereby verifying the accuracy of the theoretical model. This model effectively predicts the residual magnetic field drift law of the magnetic shielding system under varying temperature conditions and can provide theoretical support for the analysis and active compensation of thermally induced magnetic errors in atomic gyroscopes.

## 1. Introduction

The atomic gyroscope, regarded as the fourth generation of inertial sensors following mechanical, optical, and MEMS gyroscopes, holds significant application prospects in fields such as strategic-grade inertial navigation due to its high theoretical precision and exceptional sensitivity [1,2,3,4]. Among them, quantum gyroscopes represented by nuclear magnetic resonance gyroscopes and spin-exchange relaxation-free gyroscopes exhibit extreme sensitivity to magnetic fields. Consequently, magnetic suppression and compensation techniques have become the core bottleneck limiting their accuracy improvement. High-performance multilayer magnetic shielding systems are currently the mainstream solution for achieving a near-zero magnetic environment [5,6].

Currently, analysis methods for magnetic shielding devices mainly include analytical methods and numerical methods [7]. The analytical method, based on Maxwell’s equations, derives closed-form solutions for shielding coefficients through boundary conditions, offering advantages such as rapid computation and convenience for engineering design [8,9,10]. Early analytical models were mostly based on the assumption of linear permeability, making it difficult to accurately reflect the effects of apertures in actual structures and the nonlinear magnetization behavior of materials under weak fields. While numerical methods, such as the finite element method (FEM), can precisely simulate complex geometric structures and material nonlinearities, they are limited by high computational costs and long processing times, making them difficult to apply in dynamic analysis, parameter inversion, or real-time magnetic field compensation systems [11,12].

In practical operation, the residual magnetic field level of the magnetic shielding system is critical to the accuracy of atomic gyroscopes. The remanence primarily originates from the hysteresis effect of high-permeability soft magnetic materials. Traditional analytical models assume constant material permeability, which significantly deviates from the actual nonlinear magnetization characteristics of materials under extremely low magnetic fields [13]. To accurately describe the nonlinear behavior of materials and predict remanence, the Jiles-Atherton (J-A) model, which is known for its clear physical interpretation and high computational efficiency, has been widely adopted for modeling the magnetization processes of ferromagnetic materials. Integrating the J-A model with macroscopic electromagnetic field equations can significantly enhance the accuracy of predicting the true magnetic field distribution inside the shielding cavity [14].

However, existing models predominantly focus on studying hysteresis characteristics at room temperature or specific constant temperatures. High-precision atomic gyroscopes are often deployed in complex environments such as aerospace and shipborne applications, where they must operate across a wide temperature range. Studies indicate that the hysteresis loops of soft magnetic materials exhibit significant temperature dependence: temperature variations affect key magnetization parameters such as saturation magnetization, pinning coefficient, and shape parameters [15]. Experimental research by Sun et al. [16] shows that the initial permeability of 1J85 Permalloy decreases by 69.64% at −60 °C compared to room temperature and increases by 38.23% at 140 °C. Such significant changes in magnetic properties directly lead to variations in shielding performance. This “temperature-magnetism” coupling effect leads to drift in the residual magnetic field within the shielding cavity, thereby introducing measurement errors in gyroscopes [17].

Although some studies have explored the influence of temperature on material magnetization curves, there remains a lack of systematic theoretical analysis. In particular, a theoretical framework for incorporating temperature parameters into the J-A hysteresis model has yet to be established, making it difficult to quantitatively describe the effect of temperature on key magnetization parameters. Meanwhile, there is a lack of analytical prediction methods for residual magnetism in magnetic shielding that incorporate temperature effects, making it challenging to quantify the residual magnetic field drift under variable temperature conditions. Furthermore, experimental validation of existing models under varying temperature conditions is generally insufficient, and their prediction accuracy and generalization capability at different temperatures remain unclear.

To address the above issues, this paper presents a systematic modeling and validation of the temperature–residual magnetic field coupling mechanism in multilayer magnetic shielding systems. First, an analytical model of the static magnetic field for multilayer cylindrical magnetic shielding is established, and general formulas for calculating the radial and axial shielding coefficients are derived. Second, an improved J-A model incorporating a temperature correction factor is constructed, and a modified particle swarm optimization algorithm is employed to achieve global identification of model parameters at multiple temperatures. The accuracy of the proposed model is cross-validated through finite element simulations and experiments. The core contribution of this paper lies in establishing a physically driven, continuous temperature–residual magnetic field model, rather than fitting parameters at discrete temperature points. The overall research is centered on theoretical modeling, supplemented by physical experiments conducted within the actual operating temperature range of atomic gyroscopes for auxiliary validation.

This work systematically characterizes the influence of temperature on the magnetic property variations in shielding materials, providing a theoretical basis for the magnetic shielding design and magnetic error compensation of atomic gyroscopes under varying temperature conditions.

## 2. Theoretical Modeling of the Residual Magnetic Field in Magnetic Shielding Systems

**Figure 1:**
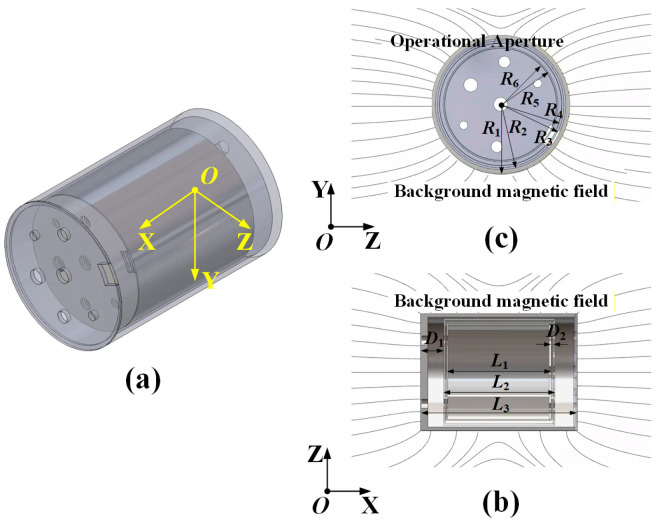
Geometric parameters and coordinate relationship of the magnetic shielding system. (**a**) Three-dimensional overall structure, (**b**) Axial cross-sectional structure, (**c**) Radial cross-sectional structure.

The research object of this paper is a three-layer cylindrical 1J85 Permalloy magnetic shielding cylinder. Modeling and analysis are carried out based on the assumption of a uniform external static magnetic field. Figure 1 shows the geometric structure, coordinate system, and key dimensions of the system. Table 1 lists all detailed geometric parameters, which are derived from the engineering CAD drawings of this project and the measurement results of the physical prototype.

The magnetic shield utilizes high-permeability materials to provide a low-reluctance path for magnetic field shielding. The static shielding factor is defined as the ratio of the external magnetic field to the magnetic field within the shielded region, and is categorized into the radial shielding factor *S^T^* and the axial shielding factor *S^A^*. Based on the assumption of an idealized geometry without apertures, this section first derives the computational models for the radial and axial shielding performance of the system by combining the Laplace equation with boundary conditions.

### 2.1. Calculation of Radial Shielding Performance in Magnetic Shielding Systems

The multilayer concentric cylindrical magnetic shielding cylinder studied in this paper is a typical axisymmetric structure; the Cartesian coordinate system in Figure 1c can be transformed into a cylindrical coordinate system. Under a uniform external static magnetic field, the magnetic field distribution is independent of the azimuthal angle *θ*, satisfying $\frac{\partial }{\partial \theta }=0$. Therefore, the three-dimensional Laplace equation in cylindrical coordinates can be reduced to its two-dimensional axisymmetric form as follows:

(1) $$\frac{1}{\rho }\frac{\partial }{\partial \rho }(\rho \frac{\partial u}{\partial \rho })+\frac{\partial^{2}u}{\partial z^{2}}=0$$

where *u* is the scalar magnetic potential, expressed in amperes (A), which is dimensionally equivalent to J/(A·m) or N/A; *ρ* and *z* represent the radial and axial coordinates, respectively.

For the radial magnetic field problem of a cylindrical surface under a uniform background magnetic field, the method of separation of variables is applied to obtain the solution. The general solution of the two-dimensional Laplace equation in each region of the circular cross-section can be expressed as:

(2) $$\varphi (\rho ,\theta )=C_{0}+D_{0}\ln\rho +\sum_{n=1}^{\infty }\left(A_{n}\cos n\theta +B_{n}\sin n\theta \right)(C_{n}\rho^{n}+D_{n}\rho^{-n})$$

In the radial shielding analysis of an n-layer magnetic shield cylinder, based on the distinct properties of the materials, the shielding structure divides the space into *k* = 2*n* + 1 regions. At the center of the shield cylinder, the scalar magnetic potential *φ*_1_ must be finite over the entire domain, which implies that as the center point is approached, the potential must satisfy $\varphi_{1}(0,\theta )<\infty$. Therefore, the coefficient *D*_1_ can be set to zero, and the magnetic potential in this region can be expressed as:

(3) $$\varphi_{1}=C_{1}\rho \cos\theta$$

In the region outside the shield cylinder, as the spatial variable *ρ* approaches infinity, the influence of the ferromagnetic boundaries on the magnetic field distribution becomes negligible. In this case, the scalar magnetic potential asymptotically approaches that of the background magnetic field *H*_0_, expressed as:

(4) $$\varphi_{2n+1}=(C_{2n+1}\rho +\frac{D_{2n+1}}{\rho })\cos\theta =-H_{0}\rho \cos\theta$$

On the inner and outer surfaces of the *n*-th layer of shielding material, the scalar magnetic potential must satisfy continuity across the interfaces, and the normal component of the magnetic flux density must be continuous:

(5) $$\left\{\begin{array}{l} \varphi_{m(2n-1)}(R_{(2n-1)},\theta )=\varphi_{m(2n)}(R_{(2n-1)},\theta )\\ \mu_{0}\frac{\partial \varphi_{m(2n-1)}}{\partial \rho }=\mu_{n}\frac{\partial \varphi_{m(2n)}}{\partial \rho }\\ \varphi_{m(2n)}(R_{\left(2n\right)},\theta )=\varphi_{m(2n+1)}(R_{\left(2n\right)},\theta )\\ \mu_{n}\frac{\partial \varphi_{m(2n)}}{\partial \rho }=\mu_{0}\frac{\partial \varphi_{m(2n+1)}}{\partial \rho } \end{array}\right.$$

where *μ*_0_ is the vacuum permeability, and *μ_n_* is the relative permeability of the *n*-th layer of shielding material. To simplify the initial theoretical calculation process, *μ_n_* is tentatively regarded as a constant in this section to complete the basic derivation.

For the three-layer shielding cylinder studied in this paper, the three shielding layers divide the space into seven independent regions. The scalar magnetic potentials in the corresponding regions are denoted as *φ*_m1_, *φ*_m2_, *φ*_m3_…*φ*_m7_. The boundary conditions to be satisfied are summarized in Table 2 below:

Based on the boundary conditions, recursive relationships for the coefficients *C_k_* and *D_k_* of the general solution to the Laplace equation across the seven regions can be derived as follows:

(6) $$\left\{\begin{array}{l} C_{k+1}=\frac{1}{2}[C_{k}(1+\frac{1}{\mu_{m}})+\frac{D_{k}}{r_{k}^{2}}(1-\frac{1}{\mu_{m}})]\\ D_{k+1}=\frac{1}{2}[C_{k}r_{k}^{2}(1-\frac{1}{\mu_{m}})+D_{k}(1+\frac{1}{\mu_{m}})] \end{array}\right.$$

By incorporating the boundary conditions, the recursive relations of the coefficients for the solution of the Laplace equation in each region can be derived. Based on these recursive relations, an iterative solution matrix is constructed, and finally the radial magnetic field distribution and the radial shielding coefficient *S^T^* of the three-layer magnetic shielding cylinder are obtained [18,19].

### 2.2. Calculation of the Axial Magnetic Shielding Coefficient for Magnetic Shielding Systems

The analysis of the axial shielding field is based on the superposition principle of the external magnetic field and the magnetic field generated by the surface magnetic charge density of the shielding cylinder. Since the longitudinal cross-section of the shielding cylinder along the axial direction is rectangular, which presents significant analytical complexity, this paper begins the derivation with the relatively simpler case of single-layer axial shielding. The axial magnetic field distribution of the magnetic shielding cylinder can be approximated by the superposition of the ambient magnetic field and the magnetic field produced by the surface magnetic charge density of the shielding cylinder. The magnetic scalar potential generated by these “magnetic charges” is solved through integration:

(7) $$\Phi (r)=\frac{1}{4\pi }\int_{s}\frac{\sigma (r^{\prime })}{\left|r-r^{\prime }\right|}ds$$

The surface magnetic field integral consists of three components: the cylindrical wall and the two end caps. The magnetic flux density at each point inside the material is determined based on the different paths through which magnetic flux enters the shielding material. For materials with finite permeability, the magnetic potential between the centers of the two end caps is given by integrating the magnetic field intensity along a path through the shielding material from the center of one end cap to the other. Assuming a uniform and constant background magnetic field, and defining the length-to-diameter ratio of the shielding cylinder as a=L/R, the formula for the single-layer axial shielding coefficient can be expressed as:

(8) $$S^{A}=1+S^{T}\left(\frac{2K}{1+a+\alpha a^{2}/3}\right)$$

where *S^T^* represents single-layer radial shielding coefficient, parameters *α* and *β* are determined experimentally, and the composite parameter *K* is derived from both.

Based on the derivation results of the single-layer shielding model described above and by combining the summation-multiplication structure of the multi-layer transverse shielding coefficients while introducing constraints, the general formula for the axial shielding coefficient *S^A^* of any arbitrary *n*-layer shielding structure can be extended as follows:

(9) $$\begin{array}{ll} S^{A}= & 1+\overset{n}{\underset{i=1}{\sum }}S_{i}^{A}+\overset{n-1}{\underset{i=1}{\sum }}\overset{n}{\underset{j>i}{\sum }}S_{i}^{A}S_{j}^{A}\left(1-\frac{L_{i}}{L_{j}}\right)+\overset{n-2}{\underset{i=1}{\sum }}\overset{n-1}{\underset{j>i}{\sum }}\overset{n}{\underset{k>j}{\sum }}S_{i}^{A}S_{j}^{A}S_{k}^{A}\left(1-\frac{L_{i}}{L_{j}}\right)\left(1-\frac{L_{j}}{L_{k}}\right)\\ & +\ldots +\overset{n-1}{\underset{i=1}{\prod }}S_{n}^{A}S_{i}^{A}\left(1-\frac{L_{i}}{L_{i+1}}\right) \end{array}$$

where *L_i_* represents the length of the shielding layer [20,21]. It can be observed from the expression that this formula not only achieves high computational accuracy but also aligns with fundamental physical intuition: when the lengths of any two shielding layers are equal, the two layers effectively merge into one, and the corresponding coupling term automatically reduces to zero.

## 3. Characterization of Temperature-Dependent Variation in Magnetic Properties of Materials

The above analytical model is established based on the assumption of constant permeability. However, in engineering practice, the permeability of soft magnetic materials exhibits nonlinear variations with the external magnetic field strength and the ambient temperature. Therefore, this section incorporates hysteresis theory to analyze the magnetic field equilibrium state of the magnetic shielding system, introduces the J-A model to characterize the nonlinear magnetization characteristics of the material, and further constructs a temperature-dependent magnetic parameter correction model.

### 3.1. Analysis of Magnetic Field Equilibrium State in Magnetic Shielding System and Equivalent Operating Point of Shielding Materials

**Figure 2:**
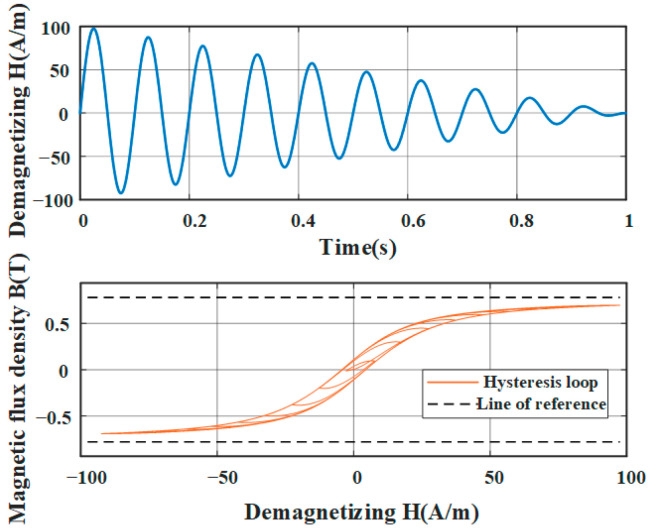
Input magnetic field strength curve and output hysteresis loop diagram of the hysteresis model.

Demagnetization is the process of applying a slowly decaying alternating magnetic field to drive the magnetic state of a material toward the anhysteretic magnetization curve. This process promotes the rearrangement of the internal magnetic domain structure until a state of zero macroscopic magnetic moment is achieved. Figure 2 shows a schematic diagram of the demagnetization process. An exponentially decaying sinusoidal waveform is used as the demagnetization excitation, with an amplitude of 100 A/m, a frequency of 10 Hz, and a total simulation duration of 1 s. The figure below presents the family of B-H hysteresis loops calculated by the J-A model under this magnetic field excitation. This figure is intended for qualitative demonstration only; the actual engineering demagnetization duration is much longer than this simulation duration.

From the perspective of magnetic domains, under saturated magnetization, the magnetic moment direction aligns with that of the external magnetic field, where the saturation magnetic moment per unit volume is represented by the saturation magnetization *M_s_*. If the volume of a single magnetic domain is *V_i_*, the corresponding magnetic moment is *M_s_*·*V_i_*. Demagnetization aims to disrupt the alignment of magnetic domains through an applied magnetic field, causing them to adopt random orientations, thereby reducing the macroscopic remanent magnetic field to zero, expressed as:

(10) $$\sum M_{s}\cdot V_{i}\cdot \cos(\theta_{i})=0$$

At this point, the material resides in a relatively balanced state, with its operating point located on the ideal anhysteretic magnetization *M_an_* curve, representing the lowest-energy stable state of the shielding material.

### 3.2. J-A Model Anhysteretic Magnetization Curve Output

Based on the framework of linear static magnetic field theory, this study further introduces the J-A model to conduct modeling and analysis of the hysteresis characteristics of shielding materials. It is assumed that after demagnetization, the shielding materials are in an ideal anhysteretic magnetization state, with their operating points located on the anhysteretic magnetization curve. Due to the gradient decrease in the magnetic field environment from the outer to inner layers in a multi-layer shielding structure, shielding materials at different positions exhibit varying magnetization states, resulting in differences in the equivalent relative permeability of each layer. The J-A hysteresis model can accurately simulate the nonlinear magnetization states of shielding materials, thereby obtaining the precise equivalent relative permeability of each shielding layer under the actual operating magnetic field.

The J-A model is based on the following physical assumptions: the magnetization process consists of two components—anhysteretic magnetization (reversible) and hysteresis loss (irreversible)—and introduces reversible magnetization *M_rev_* to describe elastic deformation. The total magnetization *M* of the material can be decomposed as:

(11) $$M=M_{an}-\delta k(dM_{\mathrm{irr}}/dH_{e})$$

where *M_an_* represents the anhysteretic magnetization intensity, describing the magnetization process of the material under an ideal equilibrium state. *M_irr_* denotes the irreversible magnetization, which accounts for the hysteresis loss component. The anhysteretic magnetization *M_an_* is described using the Langevin function:

(12) $$M_{\mathrm{an}}=M_{s}\left(\coth\left(\frac{H_{e}}{a}\right)-\frac{a}{H_{e}}\right)$$

where *a* is the shape parameter related to the pinning strength of domain walls. The irreversible magnetization *M_ir_* arises from hysteresis loss, and its rate of change is determined by the energy balance equation:

(13) $$\frac{dM_{\mathrm{ir}}}{dH}=\frac{M_{\mathrm{an}}-M_{\mathrm{ir}}}{k\delta -\alpha (M_{\mathrm{an}}-M_{\mathrm{ir}})}$$

where *k* is the hysteresis loss coefficient, and *δ* is the directional factor. Since *M_ir_* represents energy dissipation due to domain wall pinning, its rate of change depends on the deviation between the current magnetization state and the anhysteretic magnetization. The reversible magnetization *M_rev_* is further introduced to describe the elastic deformation of domain walls:

(14) $$M_{rev}=c(M_{an}-M_{irr})$$

where *c* is the reversible coefficient (0 < *c* < 1), representing the proportion of reversible magnetization to the total magnetization. By combining *M_irr_* and *M_rev_*, and substituting the expression for irreversible magnetization, we obtain:

(15) $$\frac{dM}{dH}=\frac{\left(1-c)(M_{\mathrm{an}}-M\right)}{k\delta -\alpha (M_{\mathrm{an}}-M)}+c\frac{dM_{\mathrm{an}}}{dH}$$

There is discussion in the academic community regarding whether the energy equation of the J-A model should include the (1 − *c*) factor. This paper adopts the most widely used form that includes the (1 − *c*) factor [22,23].

Based on the theoretical formulas described above, the solution code is implemented. In the main function, initial parameters and auxiliary functions are defined, and the *B*(*H*) or *B*(*M*) curves are computed according to the results obtained from the subfunctions. In the subfunctions, the anhysteretic magnetization curve *M_an_* is solved using an iterative implicit equation method, and the differential term d*M_an_*/d*H* is calculated using the central difference method.

When the relative permeability *μ_r_* of a material is not constant but depends on the magnetic field intensity *H* (i.e., hysteresis effects and nonlinear permeability are present), the analytical solution to the problem becomes relatively complex, requiring a self-consistent solution based on the relative permeability corresponding to the amplitude of the magnetic field experienced by the material. For ferromagnetic materials, the permeability *μ* is a function of the magnetic field *H*:

(16) B=μ(H)H

### 3.3. Extension of the Hysteresis Model to Account for Temperature Effects

Temperature significantly affects the magnetization characteristics of ferromagnetic materials. In this section, we will investigate the influence of temperature based on the fundamental parameters of the J-A model, including saturation magnetization *M_s_* and the domain wall pinning strength (reflected in parameters *a* and *k*). Based on the Weiss molecular field theory and the thermal activation mechanism of magnetic domains, this paper derives the following temperature correction formula.

As temperature rises, increased thermal agitation enhances the disorder of magnetic domains, leading to a decrease in the value of *M_s_*. In terms of physical mechanisms, this follows Bloch’s law (in the low-temperature region) and the Curie–Weiss law, dropping to zero at the Curie temperature *T_c_*. Therefore, a modified formula can be employed:

(17) $$M_{s}(T)=M_{s0}{\left(\frac{T_{c}-T}{T_{c}-T_{ref}}\right)}^{\beta }$$

where *M_s_*_0_ is the saturation magnetization at room temperature (*T*_ref_); *T_c_* is the Curie temperature of the material; *β* is the critical exponent describing the decay of saturation magnetization, which needs to be calibrated according to actual measurement results. When *β* = 0.5, the formula reduces to the classical Curie-Weiss approximation form.

The shape parameter *a* originates from the Langevin theory of paramagnetism, where thermal energy makes magnetic moments more prone to deviate from the direction of the external field. Theoretically, *a*(*T*) is proportional to temperature:

(18) $$a(T)=a_{0}\left(1+\gamma_{a}(T-T_{ref})\right)$$

where *γ_a_* is the temperature coefficient, typically positive (indicating that pinning strength decreases as temperature rises).

The pinning effect arises from the obstruction of magnetic domain walls by material defects. An increase in temperature reduces pinning strength, leading to a decrease in coercivity. *k*(*T*) represents the energy required to overcome pinning points. Since the actual temperature variation range in magnetic shielding systems is far from the Curie point, a simplified correction formula using linear attenuation is adopted:

(19) $$k(T)=k_{0}\left(1-\gamma_{k}(T-T_{ref})\right)$$

The magnetic domain coupling coefficient *α* reflects the interactions between magnetic domains. Although an increase in temperature weakens the exchange interactions between domains, leading to a slight decrease in *α*, its variation with temperature is relatively minor. To ensure the robustness of subsequent model parameter identification, the influence of temperature on *α* is neglected in this context.

Based on the corrections to the parameters described above, the temperature-corrected anhysteretic magnetization equation *M_an_* can be expressed as:

(20) $$M_{an}=M_{s}(T)\left[\coth\left(\frac{H+\alpha_{0}M}{a(T)}\right)-\frac{a(T)}{H+\alpha_{0}M}\right]$$

The final J-A differential equation can be expressed as:

(21) $$\frac{dM}{dH}(T)=\frac{\left[1-c(T)]\cdot [M_{an}(H,T)-M\right]}{k(T)\delta -\alpha (T)[M_{an}(H,T)-M]}+c(T)\frac{dM_{an}}{dH}(H,T)$$

It should be noted that the applicable range of the model in this paper is the temperature region far from the Curie temperature (200–400 K, i.e., −73.15 °C to 126.85 °C), which fully covers the actual operating temperature range of the atomic gyroscope (−40 °C to 60 °C) but does not enter the critical region of the ferromagnetic–paramagnetic transition. Therefore, the proposed model is not validated against the Curie–Weiss law, nor is it applicable to critical conditions as *T* approaches *T_c_* from below.

Integrating the modifications described above, this paper establishes a comprehensive analytical model of the “temperature-remanence” coupled magnetic field. It constructs a complete logical framework, ranging from fundamental shielding theory and nonlinear magnetization state characterization to the extension of the hysteresis model incorporating temperature effects. For the specific identified values of the parameters of the above J-A model, please refer to the parameter identification table presented later in this paper.

## 4. Parameter Identification for the J-A Model and Iterative Solution for Relative Permeability

**Figure 3:**
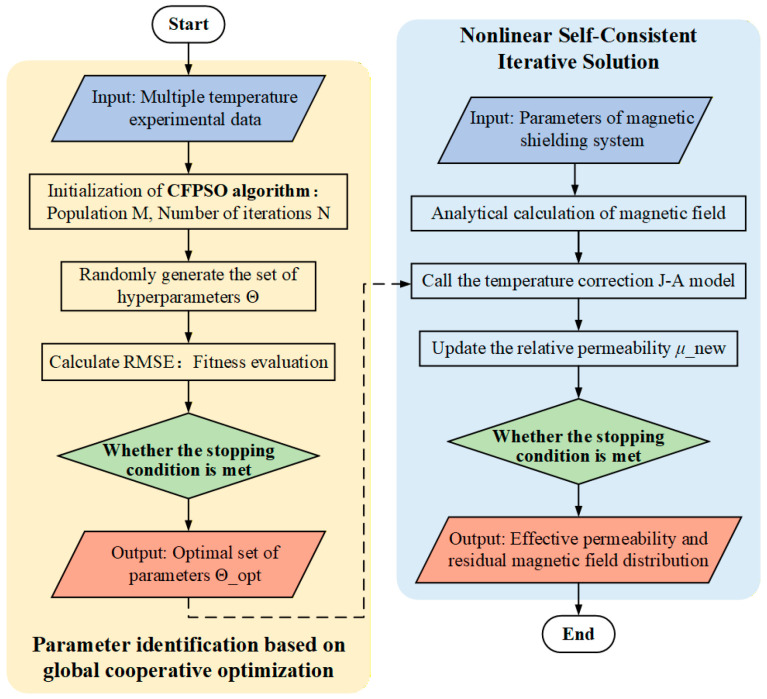
Schematic diagram of temperature correction and magnetic field iterative solution.

To address the inconsistency issues inherent in the traditional “single-point identification followed by post-processing fitting” approach, this study proposes a parameter identification strategy based on Global Synergistic Optimization. Instead of independently solving for J-A parameters at individual temperature points, this strategy integrates the temperature-dependent physical mechanisms into the parameter space, directly identifying the “hyper-parameters” that govern the evolution of the parameters. Based on the identification results, an iterative solution is performed for the equivalent relative permeability. The solution process is illustrated in Figure 3.

### 4.1. Parameter Identification for the J-A Model Based on Improved Particle Swarm Optimization

First, a generalized J-A prediction model incorporating the temperature variable *T* is constructed. The physically derived evolution equations from the earlier sections are substituted into the base model, and the parameter vector Θ to be identified is defined. Based on the derived mapping relationships, the values of each parameter at any temperature are defined accordingly. Considering model complexity and accuracy, the set of hyperparameters to be identified is defined as the following eight key coefficients:

(22) $$\Theta =[M_{s0},\beta ,k_{0},\gamma_{k},a_{0},\gamma_{a},\alpha_{0},c_{0}]$$

The mapping relationships of each parameter at any given temperature *T* are defined as follows:

(23) $$\left\{\begin{array}{l} M_{s}(T)=M_{s0}{\left[(T_{c}-T)/(T_{c}-T_{ref})\right]}^{\beta }\\ k(T)=k_{0}[1-\gamma_{k}(T-T_{ref})]\\ a(T)=a_{0}[1+\gamma_{a}(T-T_{ref})]\\ \alpha (T)\equiv \alpha_{0}\;\;\;\;c(T)\equiv c_{0} \end{array}\right.$$

Here, *T_c_* is the intrinsic Curie temperature of the material (set to 683.15 K), and *T_ref_* is the reference room temperature. Under these conditions, the magnetic flux density *B* output by the J-A model is no longer solely a function of *H*, but rather a function of *H*, *T*, and the parameter vector Θ:

(24) $$B_{sim}=f(H,T;\Theta )$$

To enable the parameter vector Θ to simultaneously satisfy the experimental characteristics at low temperature, room temperature, and high temperature, an aggregated objective function is constructed to compute the cumulative error across all tested temperature points. Assuming that *n* sets of *B*-*H* data are experimentally measured at different temperatures (with *n* = 3 defined in this study), the global root mean square error (Global RMSE) is defined as:

(25) $$J(\Theta )=\sum_{i=1}^{n}w_{i}\cdot \sqrt{\frac{1}{N_{i}}\sum_{j=1}^{N_{i}}{\left(B_{exp}(H_{j},T_{i})-B_{sim}(H_{j},T_{i};\Theta )\right)}^{2}}$$

where *i* iterates over all experimental temperatures, *j* iterates over each sampling point within a given temperature, and *ω_i_* represents the weighting coefficient, typically set to 1. If it is necessary to emphasize the fitting accuracy within a specific temperature range, the weight for that temperature group may be appropriately increased.

Given that the parameter set Θ to be optimized is high-dimensional and the objective function is highly non-convex, this study adopts a particle swarm optimization algorithm improved with a constriction factor. This algorithm effectively controls particle velocity while balancing global and local search capabilities, ensuring convergence and avoiding premature convergence. First, *M* individuals are randomly generated within a reasonable physical range, with each individual representing a set of hyperparameters Θ. These individuals are then substituted into the generalized model. The predicted curves of the model across multiple temperatures are computed in parallel, and the aggregated error *J*(Θ) is calculated as the fitness indicator. When the rate of error change falls below a threshold *ε* or the maximum number of iterations is reached, the optimal solution Θ*_opt_* is output.

**Figure 4:**
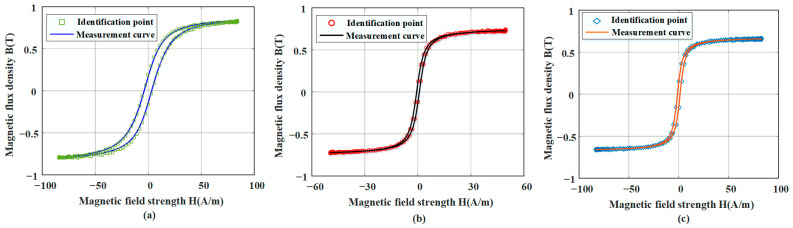
Measured curves and parameter-identified point B-H curves at three temperature points. (**a**) 200 K, (**b**) 300 K, (**c**) 400 K.

Through global synergistic identification, a unified constitutive equation describing the temperature-dependent behavior of the material can be obtained with a single optimization process. The parameters to be identified in this study include the J-A model parameters and the temperature correction coefficients. To amplify the effect of temperature on residual magnetism and thereby improve identification accuracy, we selected three temperature points with the largest span: 200 K, 300 K, and 400 K. The measured data were obtained by a third-party accredited calibration laboratory using a variable-temperature magnetic measurement system. The test samples were annular specimens of 1J85 Permalloy from the same batch, conforming to the IEC 60404-6 standard [24]. Figure 4 shows a comparison between the model-identified curves and the measured B-H data at the three typical temperatures. The identified points are in excellent agreement with the measured curves. The final identified parameters are summarized in Table 3. The temperature dependencies of the material and model parameters can be found in the preceding sections.

To further validate the generalization ability of the model, we used B-H experimental data at two intermediate temperatures, 250 K and 350 K, which were not involved in parameter identification, as an independent validation set. The results in Table 4 show that the deviations of the model-predicted saturation magnetization, coercivity, and remanence from the measured values are all less than 10%, demonstrating that the proposed model possesses the capability of continuous prediction within the temperature range.

According to mean field theory, an increase in temperature intensifies the thermal motion of lattice atoms, disrupting the orderly arrangement of magnetic moments and causing *M_s_* to follow a critical exponent law, monotonically decreasing. Macroscopically, this manifests as a compression of the height of the hysteresis loop. Simultaneously, the rise in temperature provides additional thermal activation energy *k_B_T*, assisting domain walls in overcoming energy barriers and reducing the external magnetic field energy required to overcome pinning. This leads to a significant decrease in coercivity *H_c_*. Based on Langevin statistics, an increase in temperature implies greater thermal disorder, making it more difficult for an external magnetic field to align magnetic moments coherently. This results in a gentler slope of the anhysteretic magnetization curve. The above analysis indicates that the parameter identification outcomes are consistent with physical laws, thereby validating the accuracy of the model.

### 4.2. Iterative Correction for Nonlinear Permeability

Since the permeability *μ*(*H*,*T*) of ferromagnetic materials is a nonlinear function of both magnetic field strength and temperature, and because there exists a significant magnetic field gradient from the outer to the inner layers of the magnetic shielding system, the operating points—and thus the permeabilities—differ among layers, the analytical solution based on the linear assumption cannot be directly applied. This study adopts the “Equivalent Permeability Method” combined with a fixed-point iteration strategy. Through the alternating solution of the analytical model and the J-A constitutive model, a self-consistent computation of the magnetic field distribution is achieved.

**Figure 5:**
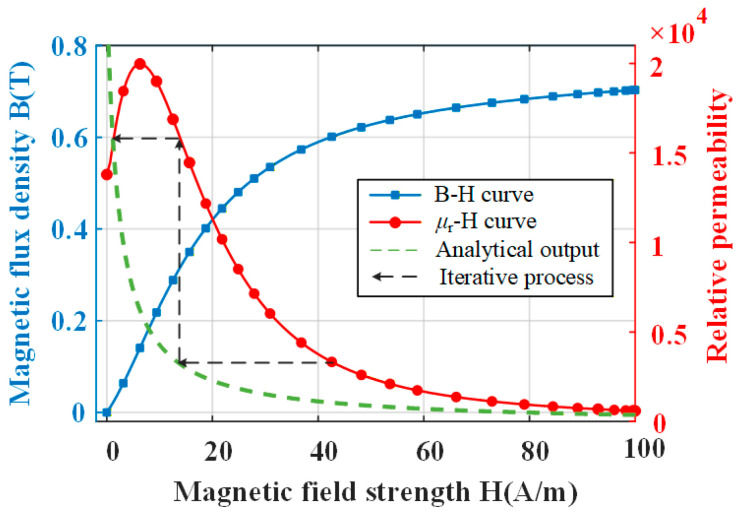
Schematic diagram of nonlinear permeability iteration.

The analytical model presented in Section 2 can solve for the magnetic field distribution in all seven regions (air regions and material layers of each layer) of the three-layer shielding structure. During the iteration process, the average local magnetic field inside each shielding layer is extracted as the input field for the J-A model, achieving data coupling between the macroscopic magnetic field and the local magnetic properties of the material. The iteration procedure in Figure 5 is as follows:

1. Initialize the equivalent permeability *μ_r_*_,*i*_(0) for each layer.
2. Substitute the permeabilities into the analytical model to solve for the magnetic field in the entire domain, and extract the average magnetic field H*_layer_*_,*i*_ for each layer.
3. Update the permeability *μ_r_*_,*i*_(1) for each layer using the *μ*-H curve generated by the J-A model.
4. Convergence criterion: The relative change in the central magnetic field of the shielding cavity is less than 1 × 10^−5^, and simultaneously the relative change in the equivalent permeability of each layer is less than 1 × 10^−4^. If satisfied, terminate the iteration; otherwise, return to step 2 to continue the calculation.

The iterative process transforms the nonlinear electromagnetic field problem into the solution of a series of linear problems until convergence and self-consistency are achieved. Upon convergence, the intersection point of the magnetic field equilibrium curve of the system and the material constitutive curve represents the effective permeability *μ_eff_* of the layer. Substituting this value into the magnetic field analysis expression yields the accurate magnetic field distribution inside and outside the magnetic shielding device, as well as within the shielding material, after accounting for material nonlinearities. All calculations in this paper are based on the static equilibrium condition after demagnetization, with the material operating on a single-valued anhysteretic magnetization curve, thus avoiding the multi-branch or multi-solution issues associated with hysteresis loops. Given the distribution characteristic of magnetic field decaying layer by layer in a multilayer structure, the approach of calculating the equivalent permeability layer by layer is well matched with the structural characteristics.

Applying the iterative algorithm, the internal magnetic field accounting for the nonlinear hysteresis characteristics of the material is calculated. The results show that compared to the linear model, the predicted magnetic field intensity at the center point under room temperature conditions increases from 0.17 nT (linear model) to 0.89 nT (nonlinear model). This indicates that the linear assumption systematically overestimates the shielding effectiveness, as it overestimates the permeability of the material in the internal low-field region. In reality, as the magnetic field in the inner layers gradually decreases, the relative permeability of the material also decreases accordingly.

## 5. Model Validation and Result Analysis

In this section, finite element simulations and physical experiments are successively employed to cross-validate the accuracy of the model under constant temperature conditions and the consistency of the model trends under varying temperature conditions.

### 5.1. Validation of Model Accuracy

FEM simulations and physical experiments are employed solely for cross-validation of the analytical model, and the predictions of the model itself do not rely on any simulation-based calibration. To validate the accuracy of the analytical model, the key geometric parameters of the three-dimensional magnetic shielding system are extracted and substituted into the analytical model for a solution. Finite element simulations are carried out using the AC/DC steady-state magnetic field module of COMSOL Multiphysics 6.2, and an ideal model without apertures and an actual engineering model considering apertures are established, respectively, for comparison.

In the FEM simulations, a fully nonlinear material model is adopted: an interpolation table of the B-H curve generated by the J-A model is defined for the 1J85 Permalloy layer. During the solution process, the equivalent permeability of each mesh element is dynamically updated according to the local magnetic field. Mesh convergence verification shows that when the mesh is refined, the change in the central magnetic field is less than 0.5%. To ensure consistency of the input magnetic field, when performing radial/axial shielding calculations, the background magnetic field amplitude is uniformly defined as 5 × 10^−5^ T (comparable to the amplitude of the Earth’s magnetic field), with the direction aligned with the calculation direction. By comparing the analytical calculation results with the numerical simulation results, cross-validation of the two methods is achieved.

The comparison results in Table 5 show that, when the shielding coefficient is calculated at the center point of the magnetic shielding system under the ideal condition without considering apertures, the analytical calculation results are in high agreement with the finite element solutions, with calculation errors of 1.3% for the radial shielding and 2.3% for the axial shielding.

In practical engineering applications, magnetic shielding systems must incorporate necessary aperture structures to meet functional requirements such as power supply and mechanical clamping. These openings inevitably affect the shielding performance of the system, necessitating a quantitative assessment of the disturbance effects caused by aperture structures on the internal magnetic field. This study systematically compares the differences in internal magnetic field distribution between an ideal aperture-free model and a practical model containing apertures. Through this comparative approach, the impact of specific structures (e.g., air gaps, apertures) on the analysis results can be accurately quantified.

First, the shielding coefficient of the static magnetic field at the center point is compared again. Table 5 shows that due to the influence of apertures, both the axial and radial shielding coefficients decrease to varying extents, with the calculation errors increasing to 3.1% and 5.2%, respectively, but still remaining at relatively low levels overall. This indicates that in multilayer magnetic shielding systems, the effect of apertures on the shielding performance is relatively small, and this effect diminishes as the number of shielding layers increases.

**Figure 6:**
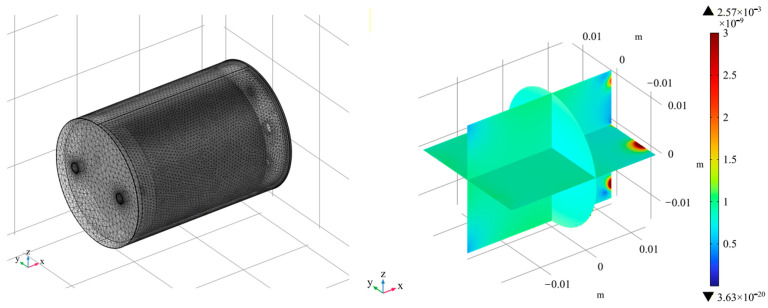
Simulation Results of Magnetic Field Considering Practical Apertures.

The FEM simulation results are shown in Figure 6. Although the influence of apertures on the shielding coefficient at the center point is weak, it is evident that the apertures disrupt the uniformity of the magnetic field and create a gradient distribution. To further verify the applicability of the analytical model, we compared the magnetic field distributions on the orthogonal cross-sections through the center of the magnetic shielding device with and without apertures, and also calculated the errors between the analytical results and the finite element results along the radial and axial lines.

**Figure 7:**
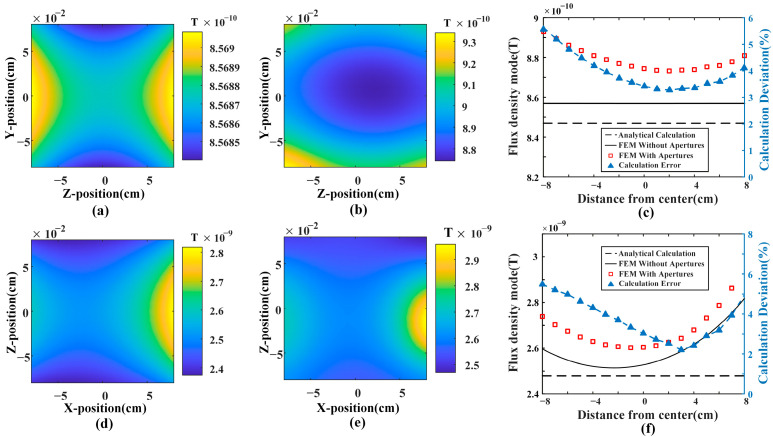
Comparison of magnetic flux density calculation results of the magnetic shielding system: (**a**) YOZ plane FEM without apertures; (**b**) YOZ plane FEM with apertures; (**c**) Comparison of analytical and FEM results along the radial line; (**d**) XOZ plane FEM without apertures; (**e**) XOZ plane FEM with apertures; (**f**) Comparison of analytical and FEM results along the axial line.

From the results in Figure 7, it can be seen that the analytical solutions and the finite element solutions are in high agreement, with the maximum deviations in the radial and axial directions (occurring near the inner wall and the end cap, respectively) both around 5%, demonstrating the accuracy of the analytical model throughout the entire spatial domain. Given that the magnetic field deviation between the actual model with apertures and the ideal model remains within a reasonable range, and through a systematic comparison of three sets of results—the ideal analytical solution, the ideal FEM solution, and the actual FEM solution with apertures—it is confirmed that, within the requirements of engineering accuracy, the ideal model can be directly adopted for magnetic shielding performance analysis.

### 5.2. Effects on the Remanence of Shielding Systems

In this section, the influence of temperature variation on residual magnetism is analyzed through a combination of simulation and experiment. Based on actual measurements, the environmental magnetic field data are summarized in Table 6. In the practical operation of atomic gyroscopes, the ambient temperature typically ranges from −40 °C to 60 °C (233.15 K to 333.15 K). To more clearly reveal the temperature dependence of the magnetic field from a theoretical perspective, we first extend the temperature range of the model calculations to 200–400 K for analysis.

**Figure 8:**
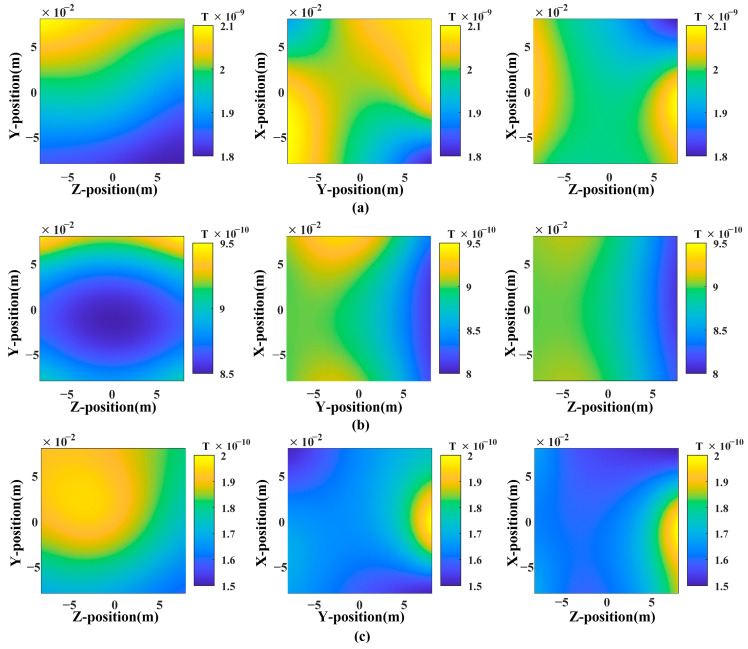
FEM simulation of the internal magnetic field distribution of the magnetic shielding system at different temperatures: (**a**) 200 K, (**b**) 300 K, (**c**) 400 K.

Within the target region of size 16 mm × 16 mm × 16 mm at the center of the magnetic shielding system, the magnetic field distributions across three cross-sections are shown in Figure 8.

Based on the extended J-A model, the hysteresis loops of the shielding material were simulated at temperatures ranging from 200 K to 400 K. The calculations show that as the temperature increases from 200 K to 400 K, the material’s saturation magnetization decreases by approximately 18%, and the coercivity decreases by 42%. Substituting this model into the system iterative calculation, it is found that the equivalent permeability of each shielding layer within the temperature range increases with rising temperature, ultimately significantly affecting the final residual magnetic field magnitude and distribution inside the system. Finite element simulation results show that the static residual magnetic field at the center point decreases from 1.97 nT to 0.18 nT, and the distribution of the residual magnetic field in the target region also undergoes considerable changes.

**Figure 9:**
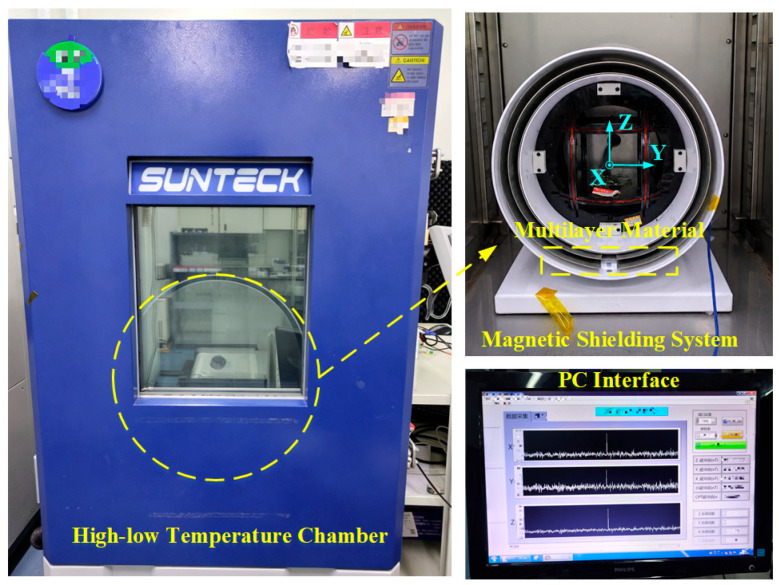
Experimental setup for the variable-temperature measurement of the magnetic shielding cylinder.

**Figure 10:**
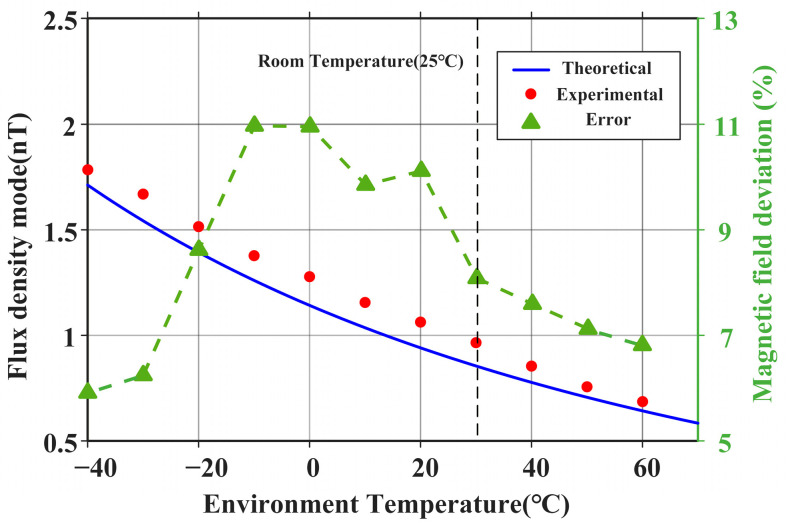
Variation curve of the static magnetic field magnitude at the center of the shielding cavity with temperature.

To validate the physical trend prediction capability of the model, we conducted residual magnetic field measurement experiments within the actual operating temperature range of the atomic gyroscope (−40 °C to 60 °C) at intervals of 10 °C. The experimental setup is shown in Figure 9. A sensor probe was fixed at the center of the shielding cavity. The magnetic shielding system was placed in a temperature chamber (Sunteck Test Equipment Co., Ltd., Shanghai, China). At each temperature point, after holding for 10 min, 10 consecutive samples were taken within 1 min and averaged. Before the experiment, the magnetic shielding cylinder was demagnetized once at room temperature; subsequent measurements at other temperature points were performed without further demagnetization to simulate actual service conditions. The experimental setup is shown in the figure. A comparison between the model predictions and the measured results is presented in Figure 10 and Table 7.

The experimental results show that the monotonic decreasing trend of residual magnetism with increasing temperature predicted by the model is completely consistent with the measured results. Within the temperature range of −40 °C to 60 °C, the relative deviation of the magnetic field magnitude is approximately 6% to 11%. As can be seen from Table 7, the deviation between the theoretical and measured axial magnetic field components is slightly larger, generally exceeding 10%, whereas the deviation for the radial component is smaller. It is believed that, on the one hand, the apertures of the magnetic shielding system are oriented along the axial direction, which has a greater influence on the analysis of the axial magnetic field component. On the other hand, this is due to the inherent properties of the magnetic shielding cylinder: the axial cross-section is rectangular, involving non-ideal factors such as edge overlaps, while the radial cross-section is circular and closer to the ideal shape. Nevertheless, the model successfully predicts the key physical trend, and its accuracy is sufficient to guide the direction of magnetic error compensation.

During the simulation, it was also found that although the absolute value of residual magnetism may decrease at high temperatures, the permeability becomes more sensitive to changes in magnetic field and temperature due to the increase in the shape parameter *a* and the decrease in *M_s_*. This means that although the residual magnetic field distribution at high temperatures is lower in magnitude, it is more sensitive to fluctuations in ambient temperature, leading to poorer long-term stability of the internal magnetic field of the shielding system. This trend is consistent with the slightly larger measured deviations observed in the high-temperature range in Table 7.

## 6. Conclusions

Traditional magnetic shielding designs often rely on assumptions of constant temperature and linear permeability, overlooking nonlinear evolutions under extreme operating conditions. This paper addresses “temperature-remanence” coupling problem in the magnetic shielding system of atomic gyroscopes by introducing a temperature-corrected J-A model, establishing a comprehensive theoretical framework from microscopic parameter identification to macroscopic field solution. The main conclusions are as follows:

1. To address the engineering bottleneck of traditional finite element analysis for multilayer magnetic shielding systems, which is time-consuming and difficult to embed into real-time compensation loops, a fast analytical model for the residual magnetic field distribution of multilayer cylindrical magnetic shielding structures is established. By introducing a nonlinear equivalent permeability iterative strategy, the model maintains the high efficiency of analytical methods while significantly reducing the overestimation error of shielding factors from traditional linear models to within 5% (1.3% in the radial direction and 2.3% in the axial direction), effectively achieving a favorable balance between computational speed and accuracy.
2. A physically driven global temperature-corrected J-A hysteresis parameter identification method is proposed, as opposed to independent fitting at discrete temperature points. By embedding the temperature-dependent variation laws of key parameters—such as saturation magnetization and pinning coefficient—into the identification framework, the model achieves continuous prediction capability across the full temperature range of 200–400 K.
3. Variable-temperature residual magnetic field measurement experiments have been conducted within the actual operating temperature range of the atomic gyroscope, with the experimental data being independent of the model construction process. The results demonstrate that the monotonic decreasing trend of residual magnetism with increasing temperature predicted by the model is in complete agreement with the measured results. The relative deviation of the radial residual magnetism ranges from 1.79% to 7.46%, and that of the axial residual magnetism ranges from 8.35% to 14.38%, confirming that the model can provide a reliable theoretical direction for magnetic error compensation.

This study refines the theoretical analysis method for magnetic shielding systems under varying temperature conditions, and holds significant academic value and engineering significance for improving the environmental robustness of high-sensitivity magnetic detection systems. Future work will focus on higher-precision in situ measurements under varying temperatures, as well as the development of active compensation strategies for the ambient magnetic field based on this model, to further enhance the overall performance of magnetic shielding systems.

## Figures and Tables

**Table 1:** Geometric parameters of the three-layer cylindrical magnetic shielding system.

Structural Component	Outer Diameter (cm)	Layer Thickness (mm)	Axial Length (cm)	Axial Spacing (cm)	Aperture Diameter (cm)
Inner layer	15.5	1.5	35.8	1.5	3.0
Middle layer	16.5	1.5	38.8	3.0	3.5
Outer layer	18.5	1.5	44.8	—	3.5

**Table 2:** Summary of Boundary Conditions.

Boundary	Boundary Conditions
1	$\varphi_{m1}(0,\theta )<\infty$
1–2	$\varphi_{m1}(R_{1},\theta )=\varphi_{m2}(R_{1},\theta )$ $\mu_{0}\frac{\partial \varphi_{m1}}{\partial \rho }=\mu \frac{\partial \varphi_{m2}}{\partial \rho }$
2–3	$\varphi_{m2}(R_{2},\theta )=\varphi_{m3}(R_{2},\theta )$ $\mu \frac{\partial \varphi_{m2}}{\partial \rho }=\mu_{0}\frac{\partial \varphi_{m3}}{\partial \rho }$
…	…
6–7	$\varphi_{m6}(R_{6},\theta )=\varphi_{m7}(R_{6},\theta )$ $\mu \frac{\partial \varphi_{m6}}{\partial \rho }=\mu_{0}\frac{\partial \varphi_{m7}}{\partial \rho }$
7	$\varphi_{m(2n+1)}(\rho ,\theta )\to \varphi_{0}(\rho ,\theta )=-H_{0}\rho \cos\theta$

**Table 3:** Identification Results of J-A Model Parameters Accounting for Temperature Effects.

Parameter	*M_s_*	*a*	*k*	*α*	*c*	*β*	*γ_a_*	*γ_k_*
Value	6.2 × 10^5^	10.4	5.1	2.2 × 10^−6^	0.6	0.4	3.3 × 10^−3^	5 × 10^−3^

**Table 4:** Model prediction and validation of material magnetic properties at intermediate temperature points.

Temperature	Physical Quantity	Measured Value	Model Predicted Value	Deviation
250 K	Saturation magnetization (A/m)	6.69 × 10^5^	6.51 × 10^5^	2.69%
	Coercivity (A/m)	1.57	1.71	8.91%
	Remanence (T)	0.51	0.47	5.40%
350 K	Saturation magnetization (A/m)	6.02 × 10^5^	5.86 × 10^5^	2.66%
	Coercivity (A/m)	1.48	1.59	7.43%
	Remanence (T)	0.37	0.35	7.8%

**Table 5:** Comparison between Analytical Calculations and Finite Element Analysis Results of Shielding Coefficients.

	Radial SF	Axial SF	Radial SF with Apertures	Axial SF with Apertures
Analysis	59,101	20,246	59,101	20,246
FEM	58,343	19,781	57,273	19,201
Error	1.3%	2.3%	3.1%	5.2%

**Table 6:** Real Background Magnetic Field Environment.

	*X*-Axis/nT	*Y*-Axis/nT	*Z*-Axis/nT
Magnetic Field	16,028	13,140	−21,413

**Table 7:** Comparison of model-predicted and experimentally measured radial/axial residual magnetism in the operating temperature range of the atomic gyroscope.

Temperature (°C)	Theoretical Axial Field (nT)	Measured Axial Field (nT)	Axial Field Deviation (%)	Theoretical Radial Field (nT)	Measured Radial Field (nT)	Radial Field Deviation (%)
60	0.59	0.64	8.47	0.27	0.29	7.41
50	0.63	0.68	7.94	0.32	0.33	3.12
40	0.69	0.77	11.59	0.36	0.37	2.78
30	0.76	0.87	14.47	0.39	0.42	7.69
20	0.84	0.96	14.29	0.43	0.46	6.97
10	0.92	1.04	13.04	0.47	0.50	6.38
0	1.02	1.15	12.75	0.52	0.56	7.69
−10	1.12	1.24	10.71	0.58	0.60	3.45
−20	1.24	1.36	9.68	0.64	0.66	3.13
−30	1.37	1.50	9.49	0.71	0.73	2.82
−40	1.50	1.64	9.33	0.78	0.75	3.85

## Data Availability

The data presented in this study are available on request from the corresponding author.
